# Pharmacokinetic study on the co-administration of abemaciclib and astragaloside IV in rats

**DOI:** 10.1080/13880209.2022.2125539

**Published:** 2022-10-13

**Authors:** Sen Sun, Lu Liu, Hongming Song, Hong Li

**Affiliations:** a Department of Anesthesiology, Shanghai Pulmonary Hospital, Shanghai, China; b Department of Endocrine, Seventh People’s Hospital of Shanghai University of Traditional Chinese Medicine, Shanghai, China; c Breast Disease Center, The Affiliated Hospital of Qingdao University, Qingdao, China; d Department of Endocrinology and Metabolism, Shanghai Tenth People’s Hospital, Tongji University School of Medicine, Shanghai, China

**Keywords:** Combined administration, metabolism, CYP3A4, transport, P-gp

## Abstract

**Context:**

The co-administration of abemaciclib and astragaloside IV might occur in the treatment of breast cancer.

**Objective:**

This study evaluates the interaction between abemaciclib and astragaloside IV in rats and describes the potential mechanism.

**Materials and methods:**

Male Sprague Dawley rats were randomly divided into four groups: single dose of abemaciclib (control), abemaciclib + 50 mg/kg/d astragaloside IV, abemaciclib + 100 mg/kg/d astragaloside IV, and abemaciclib + 150 mg/kg/d astragaloside IV. Abemaciclib and astragaloside IV were orally administrated, and astragaloside IV was pre-administrated for 7 d in the co-administrated groups. The pharmacokinetics and transport of abemaciclib were assessed in the absence or presence of astragaloside IV. In mechanism, the activity of CYP3A4 was estimated in human liver microsomes in the presence of astragaloside IV.

**Results:**

Astragaloside IV significantly increased the C_max_ (from 991.5 ± 116.99 up to 2308.5 ± 55.29 μg/L) and AUC (from 24.49 ± 2.86 up to 66.14 ± 1.17 μg/mL × h) and prolonged the t_1/2_ (from 19.85 ± 4.65 up to 66.17 ± 28.73 h) of abemaciclib, and the effect was enhanced with the increasing astragaloside IV concentration. Astragaloside IV also suppressed the transport of abemaciclib with the efflux ratio decreasing to 1.35. Astragaloside IV suppressed the activity of CYP3A4 with an IC_50_ value of 21.78 μM.

**Discussion and conclusions:**

The co-administration of abemaciclib and astragaloside IV induced the increasing systemic exposure of abemaciclib through the inhibition of CYP3A4. Further clinical validations could be carried out according to the study design of the present investigation.

## Introduction

Abemaciclib is an inhibitor of cyclin dependent kinase (CDK) 4/6, which has been applied in the treatment of breast cancer and has been studied for its effect on other malignant tumours, such as non-small cell lung cancer (Patnaik et al. 2016; Braal et al. 2021). According to the previous data, abemaciclib possesses a high efficiency in inhibiting HR^+^/HER2^–^ metastatic breast cancer and it is the only clinical drug that could be used alone (Goetz et al. 2017). A previous clinical trial reported that abemaciclib could significantly prolong patients’ overall survival but was accompanied by some adverse reactions, such as diarrhoea, nausea, and reduction of neutrophils (Dickler et al. 2017; Corona and Generali 2018). With the development of drug co-administration, abemaciclib has been combined with other treatments, such as endocrine therapy or other drugs with similar indications (Sledge et al. 2017; Johnston et al. 2020).

Traditional Chinese medicine has been widely used in clinic, especially in China. Except for cancer patients, some people received herbal treatments for anticancer therapy also increase the possibility of drug-drug interactions (Qi et al. 2015; Wang et al. 2020). *Astragalus membranaeus* (Fisch.) Bge. (Fabaceae) is a Chinese traditional medicinal plant with various pharmacological functions and has been used in the clinical treatment of breast cancer (Fu et al. 2014; Dong et al. 2022). As one of the most active ingredients of astragalus, astragaloside IV has been reported to suppress metastasis and tumour progression and enhanced the chemosensitivity of breast cancer (Jiang et al. 2017; Zheng et al. 2019; Hu et al. 2021). It is prescribed to breast cancer patients who received therapy of abemaciclib and influenced the pharmacokinetics and pharmacology of abemaciclib.

The adverse interaction between drugs and herbs is common in the clinic, which is also an important part considered in doctors’ orders. In the medical instruction of abemaciclib, the potential drug-drug interaction was taken into consideration. Some strong CYP3A4 inhibitors or inducers, such as verapamil, phenytoin, and ketoconazole, were recommended not to be combined with abemaciclib (Chong et al. 2020). The inhibitory effect of astragaloside IV on the activity of ACYP3A4 has been demonstrated in previous studies (Shan et al. 2012), and it has been reported to induce interaction with other drugs or herbs in the presence of their co-administration. However, there is little data available for the co-administration of abemaciclib and astragaloside IV.

This study focussed on the co-administration of astragaloside IV and abemaciclib in rats. With the evaluation of abemaciclib pharmacokinetic profile, abemaciclib transport, and CYP3A4 activity, the interaction between astragaloside IV and abemaciclib and the underlying mechanism was assessed.

## Materials and methods

### Chemicals and reagents

Abemaciclib (purity >98%), astragaloside IV (purity >98%), and carbamazepine (internal standard, purity >98%) were purchased from the National Institute of the Control of Pharmaceutical and Biological Products (Beijing, China). Acetonitrile and methanol (HPLC grade) were purchased from Fisher Scientific (USA). Other chemicals were analytical grade or better purchased from Sigma Aldrich (USA). Milli-Q water was filtered by the Millipore purification system and was used to dissolve chemicals and reagents.

### Study design

Male Sprague Dawley rats (Sino-British Sippr/BK Lab Animal Ltd, China) were adopted in this study. The rats were maintained under 23 ± 2 °C with a humidity of 50 ± 10%. The rats had free access to water and food but were fasted for 12 h before the experiment.

**Figure 1.**
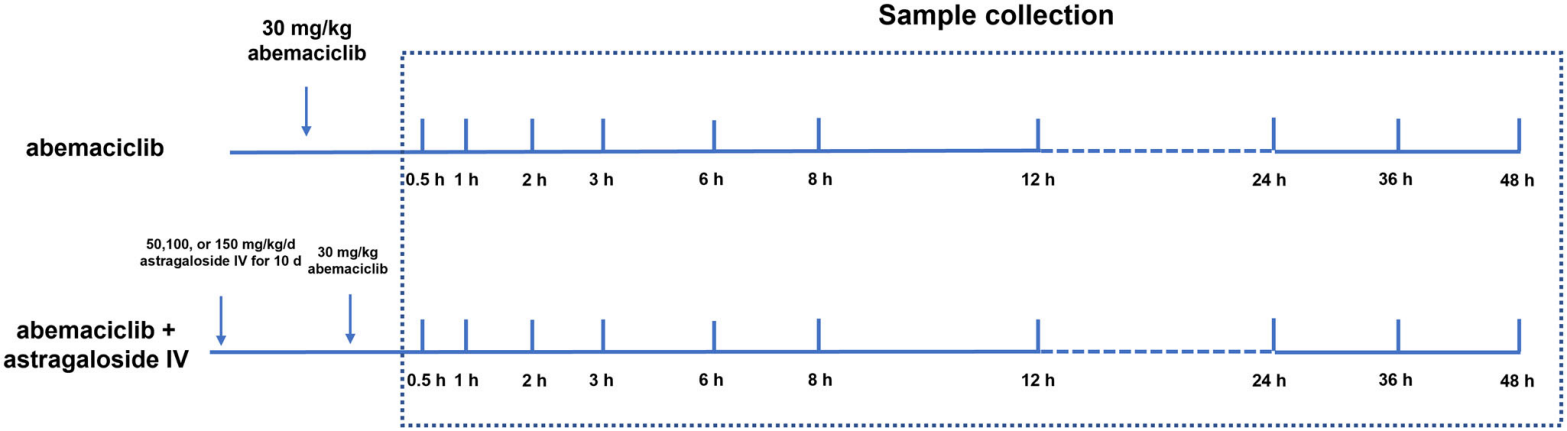
The treatments of the rats in different groups. The rats in the abemaciclib group were administrated with 30 mg/kg abemaciclib. The rats in the abemaciclib + astragaloside IV group were first administered 50, 100, and 150 mg/kg/d astragaloside IV for 10 d followed by the administration of 30 mg/kg abemaciclib. The plasma samples were collected after 0.5, 1, 2, 3, 6, 8, 12, 24, 36, and 48 h of the administration of abemaciclib.

The rats were randomly grouped into: a single dose of abemaciclib (30 mg/kg) and co-administration of abemaciclib (30 mg/kg) with astragaloside IV (50, 100, and 150 mg/kg). The specific treatment of the rats in each group is shown in Figure 1. The dosage of abemaciclib and astragaloside IV were selected according to previous studies and gavage administrated into rats for a certain period (Song et al. 2014; Dhakne et al. 2020; Gao et al. 2020).

This experiment was conducted with the approval of the Animal Ethics Committee of Shanghai Pulmonary Hospital (No.202000058). All animal experiments in this study were in strict accordance with the protocols stated in the Guide for the Care and Use of Laboratory Animals published by the US National Institutes of Health. Appropriate measures were taken to minimize the number and suffering of animals.

### Analysis of plasma abemaciclib

The blood samples were collected after 0.5, 1, 2, 3, 6, 8, 12, 24, 36, and 48 h of dosing. The collected samples were centrifugated at 3500 rpm for 10 min to obtain the supernatant and stored at −80 °C for further analysis.

The plasma concentration of abemaciclib was analyzed by the LC-MS/MS method with the Agilent 1290 series liquid chromatography system and the Agilent 6460 triple-quadruple mass spectrometer (Agilent Technologies, USA). The reaction conditions were conducted according to previous reports (Naz et al. 2018). Briefly, the samples were separated on the C18 column with the mobile phase (0.1% formic acid in water: acetonitrile). The temperature of the column was 25 °C with a flowing rate of 0.4 mL/min and an injection volume of 5 μL. The MRM mode was conducted with the collision energy of 30 eV. The MS/MS conditions were as follows: fragmentor, 110 V; capillary voltage, 3.5 kV; nozzle voltage, 500 V; nebulizer gas pressure (N_2_), 40 psig; drying gas flow (N_2_), 10 L/min; gas temperature, 350 °C; sheath gas temperature, 400 °C; sheath gas flow, 11 L/min.

The pharmacokinetics of abemaciclib was evaluated with corresponding parameters, including the area under the curve (*AUC*), half-life (*t_1/2_*), the maximum concentration (C_max_), the time reached C_max_ (T_max_), and the clearance rate (ClzF).

### Evaluation of abemaciclib transport in Caco-2 cells

Caco-2 cells were obtained from the ATCC and maintained in DMEM culture medium with 10% FBS and 1% penicillin and streptomycin. The cultured cells (1 × 10^5^ cells/cm^2^) were seeded into the transwell polycarbonate insert filters and incubated for 21 d. The culture medium was replaced every two days for the first 7 days and then daily. The transepithelial electrical resistance over 400 Ω/cm^2^ and the integrity evaluated by the paracellular flux of Lucifer yellow less than 1%/h indicated the availability of the monolayers. After the monolayers formed, it was washed with Hanks’ balanced solution and incubated for an additional 20 min. Then, abemaciclib was added to the apical or basolateral side of the monolayers and incubated in the fresh culture medium for 30 min. Astragaloside IV or verapamil was added to both the apical and basolateral sides of the monolayers. After preincubation of 30 min, the permeability of abemaciclib was evaluated in two directions (from apical to the basolateral and the opposite direction). The activity of *P-gp* was evaluated by the transport of digoxin, a typical substrate of *P-gp*. The efflux ratio over 8 was available for the transport assay.

The apparent permeability coefficient (*P_app_*) was calculated with the following equation:

$$P_{app}=\left(\Delta \text{Q}/\Delta \text{t}\right)\times \left[1/\left(\text{A}\times {\text{C}}_{0}\right)\right]$$

Where ΔQ/Δt (μmol/s) is the rate when abemaciclib appears in the receiver chamber; C_0_ (μmol/L) represents the initial concentration of abemaciclib in the donor chamber; and A (cm^2^) is the surface area of the cell monolayer.

### Evaluation of abemaciclib metabolic stability

The rat liver microsomes were incubated with abemaciclib on ice. Before the reaction, there was a preincubation of 5 min with or without astragaloside IV, and then the reaction was initiated by an NADPH-generating system. Ice-cold acetonitrile was added to terminate the reaction after a certain period. The concentration of abemaciclib was analyzed by LC-MS/MS and the metabolic stability was assessed according to its half-life and intrinsic clearance calculated using the following equations:

$$t_{1/2}=0.693/k;$$

V (μL/mg) = volume of incubation (μL)/protein in the incubation (mg);
Intrinsic clearance (Clint) (μL/min/mg protein) = V × 0.693/*t_1/2_*.

### Evaluation of CYP3A4 enzyme activity

The specific substrate of CYP3A4, testosterone (50 μM), was mixed with human liver microsomes (concentration of protein at 0.5 mg/mL), an NADPH generating system (1 mM NADP^+^, 10 mM glucose-6-phosphate, 1 U/mL glucose-6-phosphate dehydrogenase, and 4 mM MgCl_2_), and PBS buffer (100 mM, pH 7.4) in a final volume of 200 μL. The concentration of astragaloside IV was 0, 5, 10, 15, 25, 50, and 100 μM to evaluate the value of IC_50_. The reaction system was preincubated for 3 min at 37 °C in the absence of the NADPH generating system, which was added to initiate the reaction. The acetonitrile was used to terminate the reaction and the mixture was centrifugated at 12,000 rpm for 10 min. The supernatant was used for the HPLC analysis to assess the metabolites and the CYP3A4 activity.

### Statistical analysis

The pharmacokinetics of abemaciclib was analyzed with the DAS 3.0 pharmacokinetic software. The difference between groups was estimated with Student’s *t*-test. The IC_50_ value was calculated with linear regression analysis using % inhibition and log concentration by Graphpad Prism 7.0. The statistical significance was represented by *p* < 0.05.

## Results

### Effect of astragaloside IV on the pharmacokinetics of abemaciclib in rats

**Figure 2.**
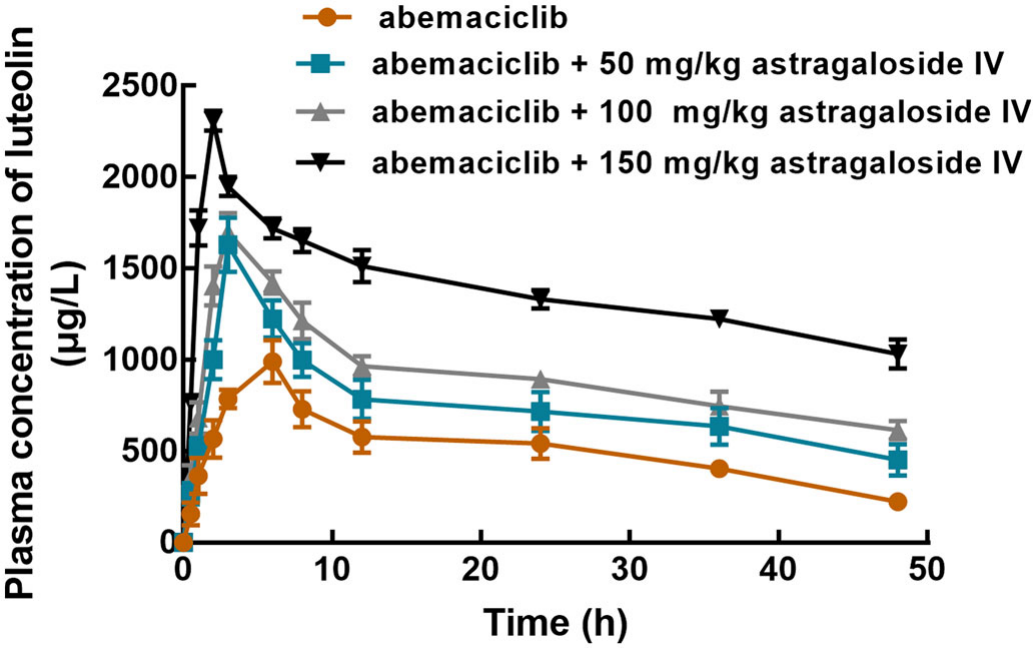
The plasma concentration-time curve of abemaciclib in the presence of 0, 50, 100, and 150 mg/kg astragaloside IV.

The pharmacokinetic profile of abemaciclib in the absence or presence of astragaloside IV is shown in Figure 2, and corresponding parameters were summarized in Table 1. In the absence of astragaloside IV, the C_max_ of abemaciclib was 991.5 ± 116.99 μg/L reached at 5.47 ± 3.24 h with the AUC of 24.49 ± 2.86 μg/mL × h. While astragaloside IV dramatically increased the C_max_ of abemaciclib to 1630 ± 147.85 (50 mg/kg), 1698.17 ± 104.43 (100 mg/kg), and 2308.5 ± 55.29 μg/L (150 mg/kg), with the increasing AUC of 36.38 ± 4.23, 43.81 ± 1.85, and 66.14 ± 1.17 μg/mL × h, respectively. Additionally, the t_1/2_ of abemaciclib was prolonged from 19.85 ± 4.65 h to 38.24 ± 7.53, 51.59 ± 17.08, and 66.17 ± 28.73 h when co-administrated with 50, 100, and 150 mg/kg astragaloside IV, respectively. The clearance rate of abemaciclib was 0.96 ± 0.067 L/h/kg under its single administration, which was significantly reduced with the increasing concentration of astragaloside IV to 0.49 ± 0.107 (50 mg/kg), 0.34 ± 0.059 (100 mg/kg), and 0.19 ± 0.042 L/h/kg (150 mg/kg). It is worthy to note that the effect of astragaloside IV was found to enhanced with increasing concentrations.

**Table 1.** The pharmacokinetic parameters of abemaciclib in the presence of various concentrations of astragaloside IV.

	Abemaciclib (30 mg/kg)	Abemaciclib (30 mg/kg) + astragaloside IV (50 mg/kg)	Abemaciclib (30 mg/kg) + astragaloside IV (100 mg/kg)	Abemaciclib (30 mg/kg) + astragaloside IV (150 mg/kg)
AUC (μg/mL × h)	24.49 ± 2.86	36.38 ± 4.23	43.81 ± 1.85	66.14 ± 1.17
C_max_ (μg/L)	991.5 ± 116.99	1630 ± 147.85	1698.17 ± 104.43	2308.5 ± 55.29
T_max_ (h)	5.47 ± 3.24	3.24 ± 1.15	2.65 ± 0.77	2.12 ± 0.84
MRT (h)	20.02 ± 0.43	20.57 ± 0.92	20.87 ± 0.40	21.76 ± 0.32
CLz/F (L/h/kg)	0.96 ± 0.067	0.49 ± 0.107	0.34 ± 0.059	0.19 ± 0.042
t_1/2_ (h)	19.85 ± 4.65	38.24 ± 7.53	51.59 ± 17.08	66.17 ± 28.73

Consistently, it was found that the half-life of abemaciclib in rat liver microsomes was 35.46 ± 10.71 min, and the intrinsic clearance rate was 39.09 ± 7.65 μL/min/mg protein. While the co-administration with astragaloside IV prolonged the in vitro half-life of abemaciclib to 44.75 ± 13.16 min with the intrinsic clearance rate of 30.97 μL/min/mg protein (data not shown), indicating the enhanced metabolic stability of abemaciclib by astragaloside IV.

### Effect of astragaloside IV on the transport of abemaciclib in Caco-2 cells

**Figure 3.**
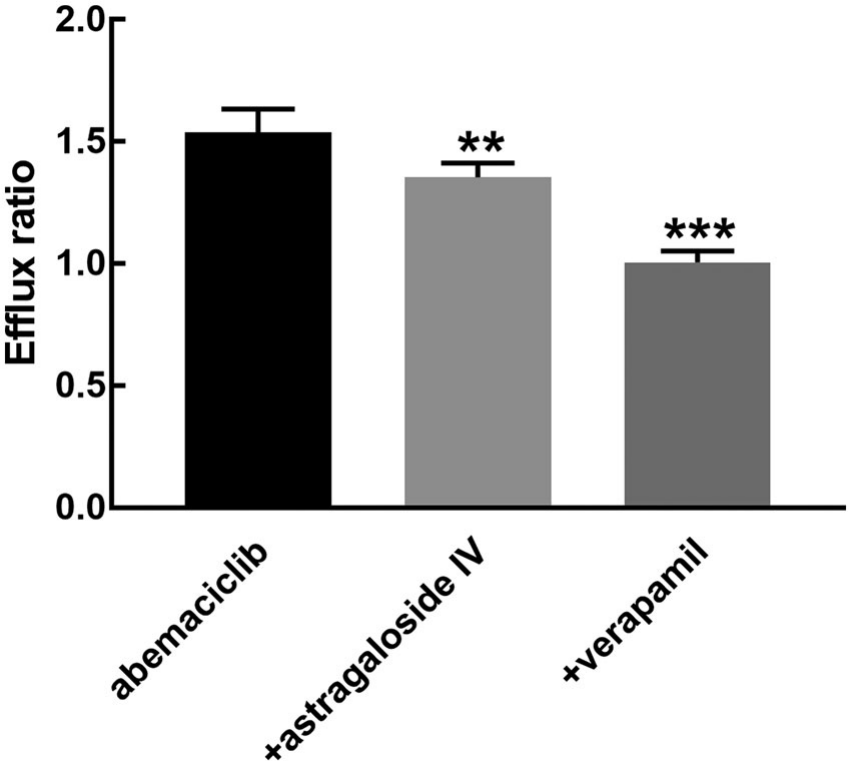
The efflux ratio of abemaciclib in the Caco-2 cells in the presence of astragaloside IV or verapamil.

In Caco-2 cells, the efflux ratio of abemaciclib was 1.54, which was dramatically suppressed to 1.01 in the presence of verapamil (*p* < 0.001, Figure 3). While astragaloside IV also showed a significant inhibitory effect on the transport of abemaciclib with a decreased efflux ratio of 1.35 (*p* < 0.01, Figure 3).

### Effect of astragaloside IV on the activity of CYP3A4

**Figure 4.**
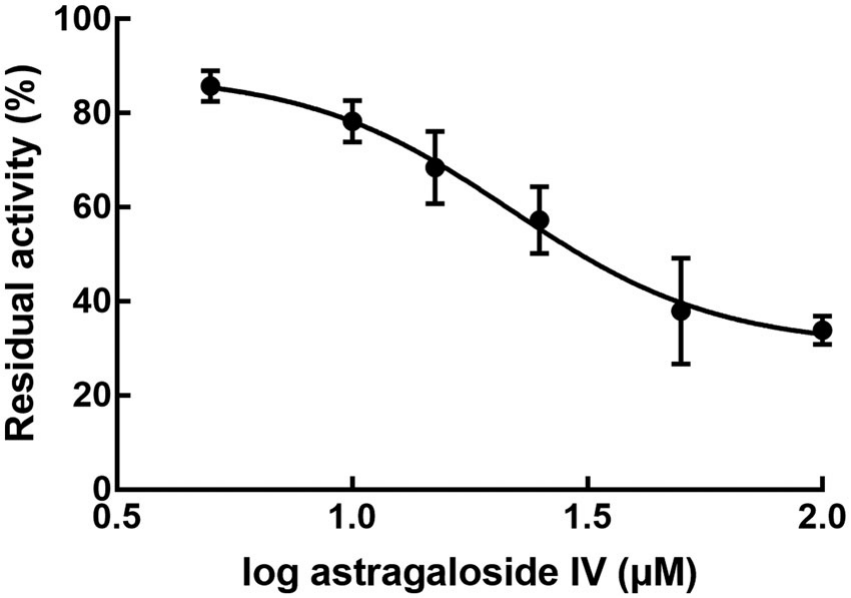
Effect of astragaloside IV on the activity of CYP3A4. To evaluate the concentration-dependent characteristics of CYP3A4 inhibition, the experiment was conducted in the presence of 0, 5, 10, 15, 25, 50, and 100 μM astragaloside IV in the pooled human liver microsomes.

In the presence of astragaloside IV, the activity of CYP3A4 was significantly reduced with the concentration of astragaloside IV (Figure 4). The IC_50_ value of astragaloside IV was 21.78 μM (Figure 4).

## Discussion

With the development of breast cancer therapy, the clinical application of abemaciclib has been considered a commonly used means due to its significant inhibitory effect on CDK4 and CDK6. Breast cancer patients always received several anticancer therapies before or after diagnosis. Among various antitumor treatments, traditional Chinese medicine has occupied a critical place. As the most critical component of Radix Astragali, the effects of astragaloside IV on various human cancers have been reported. For example, Xu et al. (2018) demonstrated the suppressed effect of astragaloside IV on the progression and metastasis of lung cancer and revealed the potential molecular mechanism that might be the regulation of the AMPK signalling pathway (Xu et al. 2018). The antitumor effect of astragaloside IV was also observed in ovarian cancer, gastric cancer, and colorectal cancer, where astragaloside IV markedly impeded tumour malignance and disease severity of patients (Liu et al. 2020, 2021; Wang et al. 2021). In breast cancer, astragaloside IV was reported to suppress the growth and metastasis of breast cancer cells and accelerate taxol chemosensitivity (Jiang et al. 2017; Zheng et al. 2019). Therefore, the co-administration of abemaciclib and astragaloside IV might occur, which might induce adverse interactions.

In the present study, abemaciclib was co-administrated with various concentrations of astragaloside IV in rats. The significant effect of astragaloside IV on the pharmacokinetic profile of abemaciclib was observed in all investigated concentrations of astragaloside IV. It was found that astragaloside IV dramatically suppressed the pharmacokinetics of abemaciclib, which prolonged the *in vivo* half-life and weakened the clearance rate of abemaciclib. *In vitro*, the metabolic stability was also found to be enhanced by astragaloside IV, which is consistent with the *in vivo* pharmacokinetic results. Moreover, astragaloside IV also influenced the transport of abemaciclib in Caco-2 cells and behaved as the inhibited efflux ratio. All these results indicated the interaction between abemaciclib and astragaloside IV during their combination.

Previously, the interactions between astragaloside IV and other herbs or drugs have been reported. For instance, Shi et al. (2022) disclosed that the co-administration of astragaloside IV and metoprolol led to the middle inhibition of metoprolol pharmacokinetics through regulating the activity of CYP2D6, a key enzyme responsible for metoprolol metabolism. The inhibited puerarin systemic exposure by astragaloside IV was illustrated to be a result of the inhibition of P-gp and CYP3A4 by astragaloside IV (Liu et al. 2019). It can be seen from these studies that CYP450s and P-gp are two crucial factors during the interaction between co-administrated drugs or herbs, especially for the CYP- or P-gp-metabolized drugs and the drugs possessing the ability to regulate CYP and P-gp activity. The inhibitory effect astragaloside IV on CYP2C9 and CYP3A4 has been indicated in previous study, and the inhibition of CYP3A4 has been verified in the present study with the IC_50_ value of 21.78 μM. Abemaciclib is specifically metabolized by CYP3A4 (Posada et al. 2020; Martínez-Chávez et al. 2021, 2022), therefore, the increasing systemic exposure of abemaciclib by astragaloside IV was hypothesized to result from the inhibition of CYP3A4. On the other hand, the transport of abemaciclib is also an important part of its biotransformation, which was mediated by P-gp. Verapamil is a typical inhibitor of P-gp, meanwhile both astragaloside IV and verapamil exerted significant inhibitory effect on the efflux ratio of abemaciclib in Caco-2 cells, suggesting the involvement of P-gp during the interaction between abemaciclib and astragaloside IV. The evaluation of CYP3A4 and P-gp activity was indirect in this study. Currently, there were several studies assessed the activity of these enzymes through their protein expression level by PCR or other analyses, which can be applied in the future studies to provide direct mechanism evidence for the pharmacokinetic interaction between different drugs or herbs.

However, there are still some limitations of this study. The present results only demonstrated the changes in the pharmacokinetics and transport of abemaciclib. The pharmacokinetics of astragaloside IV is also a vital part of the drug-drug interaction, which has been evidenced to be affected by the co-administrated drugs. For example, the combination of astragaloside IV, atractylenolide I, and prim-*O*-glucosylcimifugin in the Yu-ping-feng prescription result in the elevating C_max_ and AUC of astragaloside IV in rats (Song et al. 2014). Therefore, whether the co-administration affects the pharmacokinetics of astragaloside IV should also attract special attention in future investigations.

## Conclusions

Taken together, the inhibition of abemaciclib pharmacokinetics and transport was observed during its combination with astragaloside IV, which might result from the inhibitory effect of astragaloside IV on CYP3A4 activity. Therefore, the dose of abemaciclib should be adjusted when co-administrated with astragaloside IV.
